# Inflammasomes as the molecular hub of cardiovascular-metabolic-immune comorbidity networks

**DOI:** 10.3389/fimmu.2025.1690443

**Published:** 2025-12-19

**Authors:** Xingyu Qian, Jian Sun, Fei Li, Li Xu, Xingjian Hu, Nianguo Dong, Guangzhou Li

**Affiliations:** Department of Cardiovascular Surgery, Union Hospital, Tongji Medical College, Huazhong University of Science and Technology, Wuhan, China

**Keywords:** inflammasome, NLRP3, pyroptosis, cardiovascular disease, metabolic syndrome

## Abstract

Cardiovascular, metabolic, and immune disorders intersect through inflammasome signaling, motivating the development of a unified framework for cardiovascular risk across obesity, diabetes, infection, and autoimmunity. We first outline inflammasome architecture and activation, highlighting cryo-EM evidence that NEK7 licenses NLRP3 assembly, the coupling of priming to ion-flux and oligomerization, and cross-talk with the non-canonical caspase-4/5/11 pathway that feeds forward into IL-1β/IL-18 maturation and pyroptosis. In metabolic disease, lipotoxicity, mitochondrial ROS, oxidized lipids, and crystalline cholesterol converge on NLRP3 across adipose, myeloid, and vascular compartments, driving endothelial dysfunction, plaque growth, and adverse cardiac remodeling. Immune system diseases further amplify cardiovascular injury: population-level data link autoimmunity to heightened CVD risk, while AIM2- and NLRP3-dependent axes accelerate atherogenesis and destabilize plaques, particularly in clonal hematopoiesis and after acute infectious or ischemic insults. Translationally, anti-inflammatory trials validate this biology—IL-1β blockade lowered recurrent events in CANTOS, and low-dose colchicine reduced events in chronic coronary disease—yet heterogeneity of benefit and safety signals underscores the need for precise patient selection and timing. We propose a path forward that mirrors disease chronology: dampen priming, selectively inhibit assembly, and modulate effectors (IL-1 pathway or pyroptosis). Collectively, this review integrates mechanism and medicine to position inflammasomes as actionable hubs linking metabolic dysfunction, immune dysregulation, and cardiovascular disease, and charts priorities for precise, durable prevention and therapy.

## Introduction

Cardiovascular diseases, metabolic syndrome, and immune dysfunction constitute an interwoven global public health challenge (1–3). Metabolic syndrome affects roughly one-quarter of adults and is characterized by obesity, insulin resistance, and dyslipidemia (4); these features increase the risk of atherosclerotic cardiovascular disease by about twofold (5). Autoimmune disorders such as systemic lupus erythematosus and rheumatoid arthritis confer additional cardiovascular risk: individuals with lupus have relative risks of stroke and myocardial infarction (MI) of about 2.5 and 2.9, respectively (6, 7), and patients with rheumatoid arthritis exhibit roughly 1.5-fold higher overall cardiovascular risk (8). Given these interwoven risks, a central challenge is to identify the shared molecular pathways that drive this pathology. This review provides an updated overview of inflammasome biology in the context of cardiovascular–metabolic–immune comorbidities.

At the core of this tri-system interaction lies a shared inflammatory machinery (9–12), wherein innate immune sensors recognize metabolic stress signals as danger cues and perpetuate a maladaptive immune response (13). A central player in this process is the inflammasome—a multiprotein complex that integrates pathogen-, damage-, and metabolism-associated signals to initiate inflammation (14). Beyond its classical role in host defense, the inflammasome has emerged as a molecular hub linking metabolic imbalance, immune activation, and cardiovascular pathology (15–17).

In this review we discuss how inflammasome signaling orchestrates disease-relevant immune responses across systems, and evaluate its potential as a therapeutic target for cross-disease intervention.

## Structural basis for inflammasomes as the molecular hub

Inflammasomes are intracellular multiprotein signaling platforms that integrate infectious, sterile and metabolic danger cues into a stereotyped inflammatory output (18, 19). Canonically, an inflammasome comprises a sensor molecule and an adaptor protein that recruit the effector pro-caspase-1 (20, 21). Conceptually, these assemblies behave as supramolecular organizing centers (SMOCs) of innate immunity, in which a limited set of caspase-1–dependent effector programs can be plugged into many distinct upstream pattern-recognition events. This modular design – sensor, adaptor, effector – is the structural basis for the role of inflammasomes as molecular hubs.

The sensor molecule is typically a member of the nucleotide-binding leucine-rich repeat (NLR) or AIM2-like receptor (ALR) families, while the primary adaptor protein is Apoptosis-associated speck-like protein containing a CARD (ASC), which contains both pyrin (PYD) and caspase activation and recruitment domains (CARD). Most NLR proteins possess a tripartite architecture of an N-terminal PYD or CARD, a central nucleotide-binding and oligomerization (NACHT) domain mediating oligomerization, and C-terminal leucine-rich repeats (LRRs) that act as regulatory domains (22, 23). Upon activation, sensors such as NLR Family Pyrin Domain Containing 3 (NLRP3) undergo conformational change and oligomerization, recruiting ASC and caspase-1 to form a helical filamentous structure that nucleates the assembly of a micron-sized ASC speck (24, 25). Within this scaffold, proximity-induced activation of caspase-1 leads to cleavage of pro-IL-1β and pro-IL-18 into mature cytokines and to proteolytic activation of the pore-forming protein gasdermin D (GSDMD) (26); the N-terminal fragment of GSDMD inserts into membranes to execute pyroptosis, a lytic form of cell death accompanied by cytokine release (27). The membrane translocation process was recently proved to be mediated by S-palmitoylation of GSDMD at Cys^19^ (28). Emerging evidence also implicates gasdermin E (GSDME) as a mediator linking apoptosis to pyroptosis when cleaved by caspase-3 or-8 (29, 30). Together, these features illustrate how a common ASC–caspase-1–gasdermin effector module can be engaged by multiple structurally distinct sensors.

Numerous sensor proteins can form inflammasomes. NLRP3 is the most studied and responds to diverse triggers including ionic fluxes, reactive oxygen species, crystalline substances, and metabolites (31, 32); NIMA-related kinase 7 (NEK7), a serine–threonine kinase, has been identified as an essential scaffold linking potassium efflux to NLRP3 activation (21, 33). Other NLRs include NLRP1, NLRP6, NLRP7, NLRP9, NLRP12 and NLRC4 (34, 35). Specifically, NLRP1 contains a function-to-find (FIIND) domain, which undergoes autoproteolysis to generate an N-terminal fragment and a C-terminal fragment containing a CARD and the UPA (conserved in UNC5, PIDD, and ankyrins) subdomain (36). In an inactive state, these fragments are non-covalently associated, with the FIIND domain serving as an auto-inhibitory mechanism (37, 38). Members of the pyrin and HIN domain (PYHIN) family, such as AIM2 and interferon-inducible protein 16 (IFI16), detect cytosolic double-stranded DNA (39, 40), while pyrin senses Rho GTPase modifications (41). A subset of NLR family proteins that do not typically assemble inflammasomes—such as NLRP12 and NLRX1—have been identified as regulatory NLRs (42, 43); they often dampen NF-κB signaling, thereby constraining the activation of canonical inflammasomes (44–46). These examples underscore a general principle: sensor diversity is wired into the N-terminal recognition domains, whereas signal amplification and effector execution are shared through a conserved ASC–caspase-1 module, enabling the inflammasome system to centralize multiple upstream danger inputs.

## Unifying activation principles of inflammasomes as the molecular hub

The activation of most inflammasomes follows a two-signal model that provides a unifying framework for understanding how these complexes function as molecular hubs. A priming stimulus-typically Toll-like receptor or cytokine receptor engagement activates NF-κB to up-regulate expression of NLRP3, pro-IL-1β and pro-IL-18 and to install permissive post-translational marks. A second trigger—potassium efflux through P2X7 receptor (P2X7R), calcium influx, lysosomal rupture by engulfed crystals, mitochondrial dysfunction or increased metabolic intermediates levels (e.g., succinate, cardiolipin, fatty acids)—initiates sensor oligomerization (47–49). Rather than binding a wide range of ligands directly, NLRP3 behaves as a sensor of cellular homeostatic perturbation, with heterogeneous upstream signals converging on a limited set of conserved intracellular events (50).

In the case of NLRP3, rather than binding directly to an array of ligands, activation is triggered by sensing their downstream cellular consequences. These diverse cues converge on two principal events: ionic imbalance, most critically potassium (K^+^) efflux, and mitochondrial dysfunction, which results in the production of mitochondrial ROS and the cytosolic release of mitochondrial DNA (51, 52). Disruption of intracellular ion homeostasis represents the most fundamental and unifying trigger. A drop in cytosolic potassium (K^+^) concentration is considered the canonical, final common step for nearly all activators (53). This K^+^ efflux, induced by pore-forming toxins or extracellular ATP acting on P2X7R, promotes a conformational change that renders NLRP3 competent for activation. Fluxes of other ions provide additional layers of regulation: increased cytosolic calcium (Ca²^+^) can amplify NLRP3 responses by exacerbating mitochondrial stress, whereas chloride (Cl^−^) efflux facilitates NLRP3 oligomerization (54–56). A second critical point of convergence is mitochondrial dysfunction. Metabolic stressors, such as saturated fatty acids and hyperglycemia-induced reactive oxygen species (ROS), invariably compromise mitochondrial integrity, leading to the release of mitochondrial-derived damage signals, including mitochondrial DNA (mtDNA) and mtROS (52, 57, 58). A third conserved danger module is lysosomal damage. Phagocytosed crystalline structures, such as cholesterol crystals in atherosclerosis or monosodium urate crystals in gout, destabilize lysosomes, release cathepsins and robustly trigger NLRP3 activation (59–61). Specific metabolic intermediates thereby link metabolic dysregulation to these core cellular stress signatures by inducing ER stress, mtROS production and lysosomal injury (62, 63).

The detection of stress signals alone is not sufficient for activation; a sophisticated gating mechanism ensures that the response is proportional, context-appropriate, and avoids spurious activation. This module determines the activation threshold and the sensitivity of the response in different cell types. The serine/threonine kinase NEK7 functions as an essential, non-redundant licensing factor for NLRP3 activation. Following the primary input signal of K^+^ efflux, NEK7 directly binds to the leucine-rich repeat (LRR) domain of NLRP3. This binding event acts as a critical checkpoint, sanctioning the subsequent oligomerization of the NLRP3 scaffold and linking ionic signals directly to structural rearrangement (33, 64, 65). Upon activation, NLRP3 and associated components can undergo liquid–liquid phase separation (LLPS) to form biomolecular condensates, which rapidly concentrate inflammasome proteins within a membrane-less compartment and thereby facilitate protein–protein interactions required for ASC speck formation (66). Post-Translational Modifications (PTMs) form a dynamic regulatory network that acts as a rheostat, fine-tuning the sensitivity and amplitude of the NLRP3 response (67). In resting cells, NLRP3 is often tagged with K48-linked ubiquitin chains, marking it for proteasomal degradation and keeping its levels low, a process reversed by deubiquitinating enzymes upon priming (68). Conversely, JNK1-mediated phosphorylation at serine 194 prepares NLRP3 for activation by promoting deubiquitination and self-association (69). More recently, acetylation and palmitoylation have been shown to add further precision. Acetylation at lysine 24 by lysine acetyltransferase 5 (KAT5) further enhances NLRP3 oligomerization without affecting its recruitment to the trans-Golgi network (70). ZDHHC7-mediated palmitoylation licenses NLRP3 activation by facilitating its transition into a phase-separated state upon encountering activators such as K^+^ efflux, imiquimod, palmitate and cardiolipin (70, 71). Post-transcriptional mechanisms also contribute to inflammasome regulation. For example, the N^6^-methyladenosine (m^6^A) methylation of NLRP3 mRNA, mediated by the Wilms’ tumor 1-associated protein (WTAP), has been shown to increase NLRP3 protein levels by enhancing mRNA stability (72). Arsenic (+3) methyltransferase (AS3MT) has been identified as a key facilitator of NLRP3 activation, both by physically interacting with the NLRP3 protein and by promoting the m^6^A-mediated stabilization of its mRNA transcript (73).

Noncanonical and alternative inflammasome pathways can be understood within the same unified framework. In noncanonical signaling, caspase-4/5/11 directly recognize intracellular LPS, cleave GSDMD and secondarily activate NLRP3 (74, 75). Further, alternative inflammasomes can be activated by protease cleavage or in response to the presence of cytosolic DNA, irrespective of its microbial or host origin (76, 77). In each case, diverse proximal triggers ultimately feed into the same effector module—GSDMD-dependent membrane permeabilization and caspase-1–mediated IL-1 family cytokine maturation, once passing through layers of licensing by ionic flux, organelle stress and PTMs.

In summary, three interlocking principles define inflammasome activation as a molecular hub. The first is broad input surveillance, whereby chemically unrelated stimuli are translated into a small set of conserved ionic, organellar and metabolic danger modules. The second is a tightly regulated gating and licensing system, incorporating NEK7, phase separation and multilayer PTMs modifications to set the activation threshold. The third is coupling of these inputs to a shared ASC–caspase-1–gasdermin effector module. This integrated design allows inflammasomes to respond appropriately to a vast range of infectious, sterile and metabolic threats while maintaining tissue homeostasis, thereby placing them at the core of the cardiovascular–metabolic–immune comorbidity network.

## Molecular mechanisms linking metabolic dysregulation to cardiovascular injury

Metabolic syndrome and its main components—central obesity, insulin resistance, and dyslipidemia—create a pro-inflammatory milieu that damages the vasculature and heart (78–80). Adipose tissue in obesity becomes infiltrated by pro-inflammatory macrophages, neutrophils, and T cells (81). Adipocytes and infiltrating macrophages express high levels of NLRP3 and secrete IL-1β and IL-18, driving local and systemic inflammatory response (62, 82, 83). Over time, adipocytes develop disinhibited lipolysis, releasing excess non-esterified fatty acids and accumulating bioactive ceramides; concomitantly, adipose cholesterol handling shifts toward enhanced efflux and lipoprotein remodeling (84). In the context of metabolic stress, particularly within adipose tissue and the liver, different fatty acids bidirectionally regulate the NLRP3 inflammasome. Saturated free fatty acids (e.g., palmitate) and ceramides activate Toll-like receptor 4 (TLR4) on resident immune cells like macrophages promoting ER stress and mitochondrial ROS, thereby priming and activating NLRP3 (85, 86); unsaturated fatty acids, by contrast, generally exert anti−inflammatory effects (87). Notably, ω−3 fatty acids engage GPR40 and GPR120 on adipose tissue macrophages, and through the downstream scaffold β−arrestin−2, directly bind with NLRP3 to suppress its activation (87, 88).

The pro-inflammatory microenvironment induced by obesity then activates stress kinases and transcriptional programs—most notably c-Jun N-terminal kinase (JNK) and NF-κB—culminating in inhibitory serine phosphorylation of Insulin Receptor Substrate-1 (IRS-1) and impaired propagation of insulin-receptor signaling (89, 90). Hyperglycemia further enhances mitochondrial ROS and activates the TXNIP–NLRP3 axis (91, 92). Additionally, islet amyloid polypeptide aggregates in type 2 diabetes act as crystalline activators of NLRP3 (93, 94). These metabolic stressors converge on caspase-1 to elevate IL-1β and IL-18, which impair insulin signaling in the liver, leading to a vicious circle (95).

The vasculature is directly assaulted by inflammasome-related signals. Dyslipidemia leads to the accumulation of cholesterol crystals and oxidized low-density lipoprotein (oxLDL) within arterial walls (96, 97). Cholesterol crystals rupture phagolysosomes and robustly activate NLRP3 (98, 99). Meanwhile, oxLDL engages scavenger receptors and induces ROS, promoting NLRP3 priming (100, 101). Activated macrophages produce IL-1β and IL-18, recruit monocytes and neutrophils, and destabilize plaques (102–104). AIM2 activation in macrophages also promotes necrotic core expansion and plaque instability—particularly in diabetes and clonal hematopoiesis—highlighting convergent DNA-sensing routes that intersect with NLRP3 biology and innate immune training (105–107).

Beyond immune cells, key resident cells of the cardiovascular system are also direct participants in inflammasome-driven pathology. Endothelial cells themselves also assemble NLRP3 and undergo pyroptosis in response to metabolic stressors (108, 109). Through ROS- and TXNIP-linked pathways, high glucose promotes NLRP3-dependent endothelial pyroptosis and barrier dysfunction. This compromised endothelial integrity both increases the direct uptake of lipids by endothelial cells and facilitates the passive accumulation of lipoproteins in the subendothelial space, collectively amplifying leukocyte adhesion and atherogenesis (110, 111). Metabolic stress likewise associates vascular smooth muscle cells (VSMCs) transdifferentiation with enhanced NLRP3 activity (112). In diabetes-affected hearts, NLRP3 activation in cardiomyocytes and infiltrating macrophages enlarges infarct size and accelerates maladaptive ventricular remodeling (113).

Besides lipids and glucose, mitochondrial DNA and other metabolic factors also engage inflammasomes. In metabolic steatohepatitis, hepatocyte injury releases mitochondrial DNA and ATP, activating NLRP3 in Kupffer cells (114, 115). IL-1β from liver inflammation then promotes systemic insulin resistance and atherogenesis. Other metabolic waste products and nutrients also function as potent inflammasome triggers, linking pathologies in different organ systems. For instance, homocysteine, a byproduct of methionine metabolism that accumulates in kidney dysfunction, can trigger NLRP3 activation in vascular smooth muscle cells and endothelial cells, promoting atherosclerosis (116). Similarly, uric acid, a breakdown product of purines that is elevated in both gout and obesity-related metabolic syndrome, crystallizes within macrophages and renal cells, directly activating the NLRP3 inflammasome and thereby linking metabolic dysregulation to both vascular and kidney inflammation (59). Furthermore, metabolites derived from diet and gut microbiota can contribute to systemic inflammation. Elevated circulating branched-chain amino acids (BCAAs), associated with obesity and insulin resistance, have been shown to induce mitochondrial stress and activate the NLRP3 inflammasome in macrophages (117). Concurrently, increased gut permeability in metabolic syndrome can lead to the translocation of microbial endotoxin (LPS) into circulation, which primes the inflammasome in a wide range of tissues (118).

**Figure 1:**
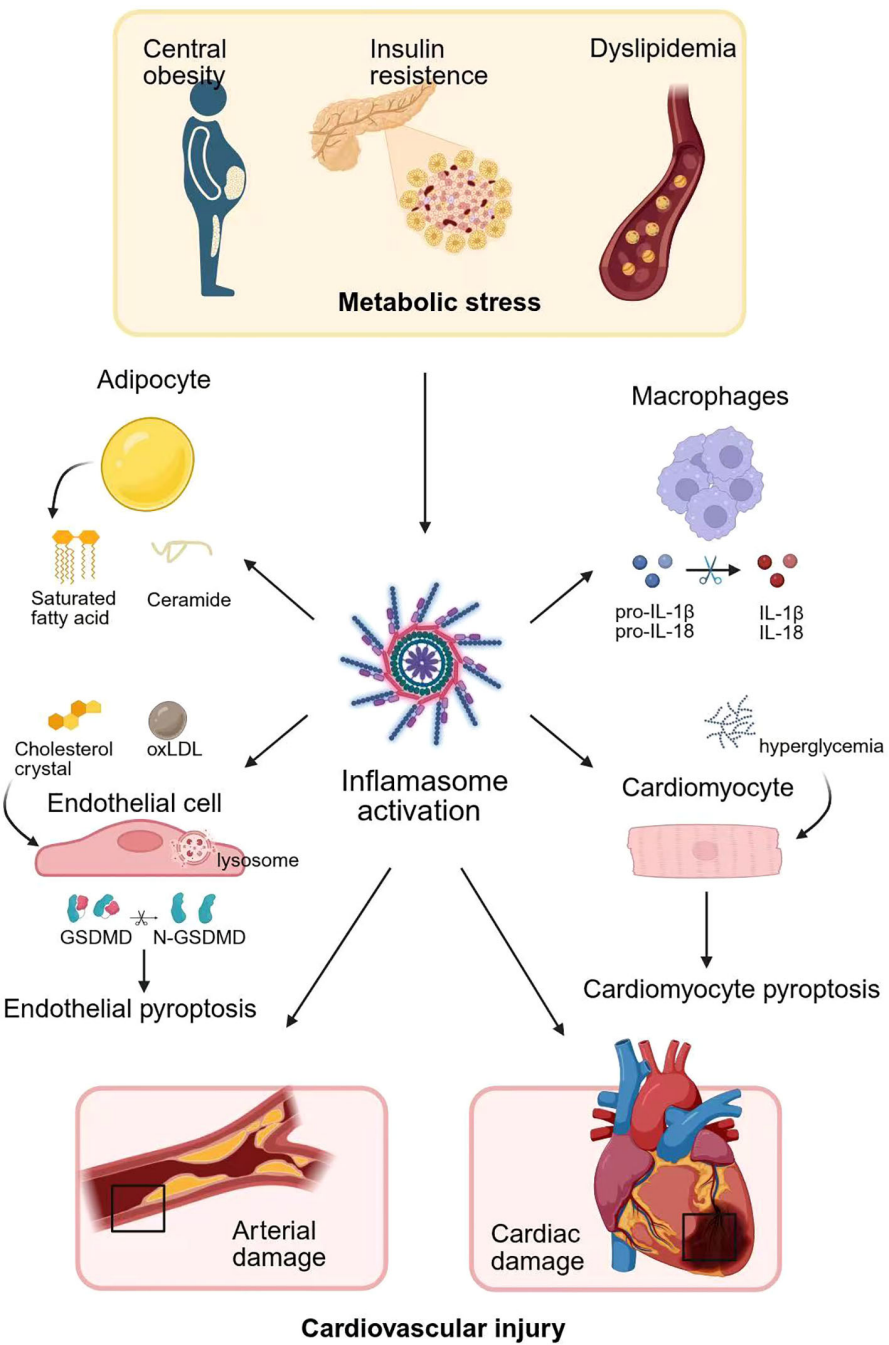
Metabolic dysregulation fuels cardiovascular injury via inflammasomes. Metabolic stress, characterized by central obesity, insulin resistance, and dyslipidemia, initiates inflammasome activation across multiple cell types, driving cardiovascular injury. In adipocytes, metabolic overload leads to lipolysis and the accumulation of saturated fatty acids and ceramides. As the principal effector cells infiltrating multiple organs, macrophages exhibit a high level of proinflammatory cytokine secretion. Cholesterol crystals and oxLDL rupture lysosomes and induced pyroptosis of endothelial cells, leading to barrier dysfunction. Similarly, metabolic stressors such as hyperglycemia can activate NLRP3 in cardiomyocytes, thereby exacerbating myocardial infarction. Collectively, these processes cause both arterial and cardiac damage, culminating in cardiovascular injury. oxLDL, oxidized low-density lipoprotein; ROS, reactive oxygen species.

Collectively, metabolic inputs prime and trigger inflammasome signaling in adipocytes, endothelium, VSMCs, macrophages, and cardiomyocytes. The resulting IL-1β/IL-18 release and pyroptotic damage form a feed-forward circuit that links metabolic syndrome to atherosclerosis, infarct expansion, and heart failure. Breaking this circuit—by correcting upstream metabolism, restoring mitochondrial/autophagic homeostasis, and selectively inhibiting inflammasome/IL-1 signaling—represents a coherent strategy to reduce cardiovascular injury in metabolic disease (**Figure 1**).

## Immune dysregulation and cardiovascular events

Acute and chronic infections deliver immune danger signals directly to the cardiovascular system. In gram-negative sepsis, intracellular lipopolysaccharide activates the noncanonical inflammasome pathway (caspase-4/5 in humans; caspase-11 in mice), cleaving gasdermin-D (GSDMD) to induce pyroptosis (74, 119) in pyroptotic myeloid and endothelial cells expose phosphatidylserine and tissue factor, amplifying immunothrombosis and driving sepsis-associated coagulopathy and multiorgan injury (120, 121).In the heart, NLRP3 inflammasome-driven septic cardiomyopathy, where the resulting IL-1β and IL-18 directly induce both myocardial depression by impairing contractility and myocarditis-like features characterized by inflammatory cell infiltration and cardiomyocyte pyroptosis (122). Recent basic and translational work tightly links this inflammasome–pyroptosis–coagulation axis to clinical phenotypes—including sepsis-induced coagulopathy (SIC), disseminated intravascular coagulation (DIC), and septic cardiomyopathy—and highlights endothelial caspase-11/GSDMD as a critical pathological node and a plausible therapeutic target (123, 124).

Viral immune assaults, exemplified by COVID-19, further underscore the link between inflammasomes and cardiovascular complications (125). The SARS-CoV-2 nucleocapsid (N) protein and viroporin-like accessory proteins (e.g., ORF3a) can license or trigger NLRP3 assembly in macrophages and epithelial/endothelial cells, leading to IL-1 family cytokine release and pyroptosis (126). These processes allow lung injury to spill over into a systemic thrombo-inflammatory state and heighten the risk of arrhythmias, myocardial injury, and ischemic events in susceptible individuals. Selective NLRP3 inhibitors have partially reversed downstream inflammatory markers: in mouse models of influenza A and SARS-CoV-2 infection, treatment with the NLRP3 inhibitor significantly reduced pulmonary levels of IL-1β and IL-18, decreased neutrophil infiltration, and attenuated acute lung injury; moreover, early clinical trials using the inflammasome inhibitor improved clinical outcomes in participants (127, 128). These findings offered a testable pathway hypothesis for targeting virus-related cardiovascular complications. Low-grade, persistent immune activation also imprints long-term cardiovascular risk. Even in the era of effective antiretroviral therapy, people living with HIV retain a 1.5–2-fold increase in cardiovascular events; experimental data suggest HIV can augment foam-cell formation and atherogenesis through NLRP3 signaling (129, 130).

A growing body of clinical evidence indicates that aberrant inflammasome activation underlies the heightened cardiovascular risk observed in autoimmune disorders (131, 132). Patients with systemic lupus erythematosus (SLE) and rheumatoid arthritis (RA), for example, exhibit clear signs of inflammasome upregulation in affected tissues and blood. Indeed, gene expression analyses of kidney biopsies from lupus nephritis patients show increased transcription of NLRP3, ASC, caspase-1, and IL-18, confirming that the inflammasome machinery is activated *in vivo* in SLE (133). Correspondingly, SLE and RA patients have markedly elevated serum IL-18 levels compared to healthy individuals (134). Notably, these high IL-18 levels correlate with impairments in vascular repair – in lupus, excess IL-18 is associated with endothelial progenitor cell dysfunction and hence may contribute directly to premature vascular damage (133). Rheumatoid arthritis shows a similar pattern of inflammasome involvement. RA synovial tissues express significantly higher levels of IL-1β and IL-18 than osteoarthritic controls, with the IL-18 protein strongly localized to infiltrating pro-inflammatory macrophages in the synovium (135). Certain genetic variants in inflammasome components have been linked to greater RA disease activity and accelerated atherosclerosis in these patients, further supporting a causal role (136). Beyond systemic markers, direct evidence in target organs reinforces the connection between inflammasomes and autoimmune cardiovascular comorbidities. Immunohistochemical studies of atherosclerotic lesions have found interleukin-18 abundantly expressed in human plaques, especially in macrophage-rich areas of unstable plaques, whereas little to no IL-18 is present in normal arterial tissue, implicating inflammasome-derived cytokines in the erosion and rupture of lesions (137). Collectively, these findings provide a direct pathophysiological link between inflammasome activation and the observed increase in cardiovascular injury in autoimmune diseases. This clinical insight reinforces the rationale for targeting inflammasome pathways to mitigate cardiovascular risk in autoimmune populations.

**Figure 2:**
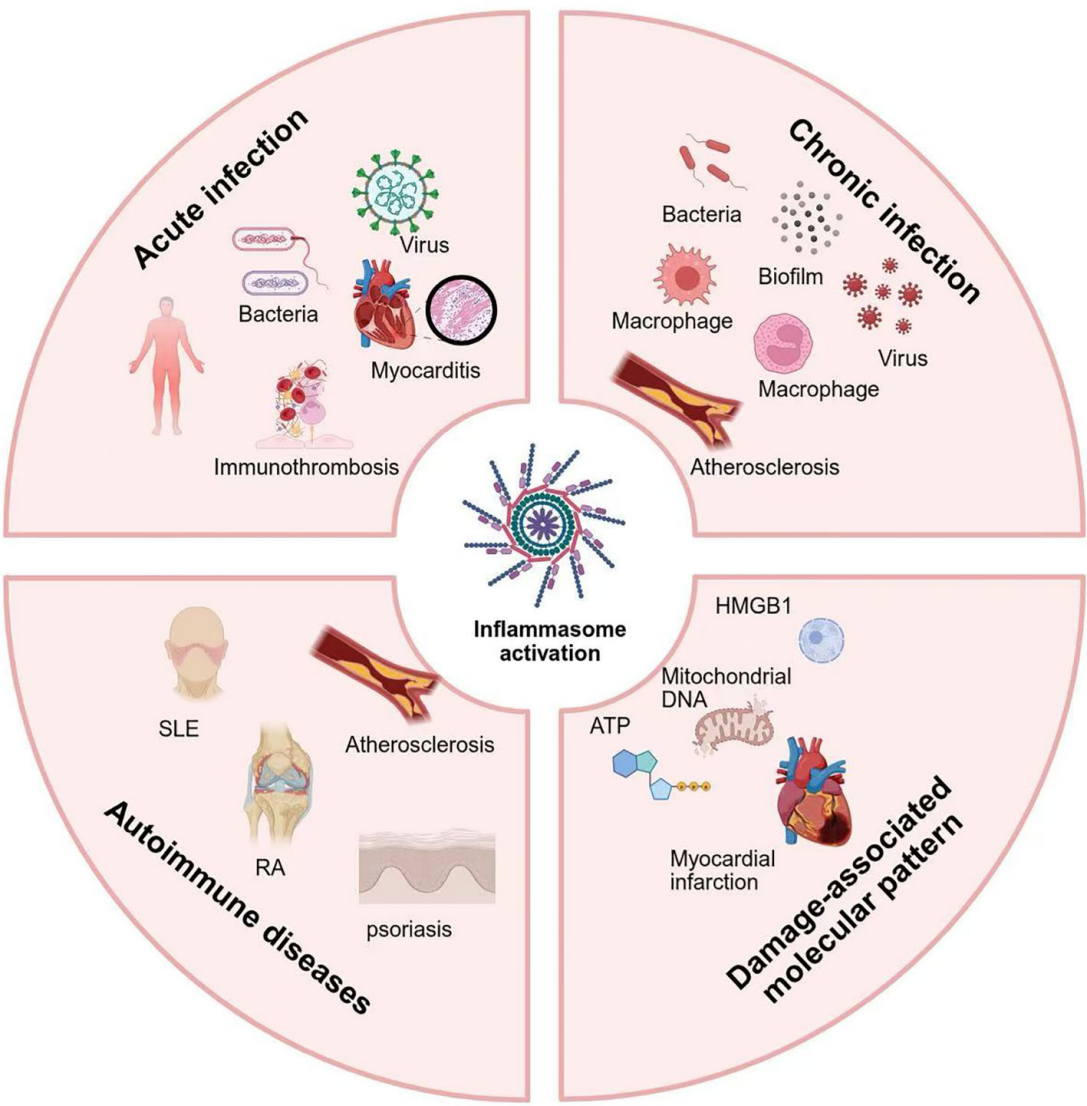
Immune dysregulation–inflammasome axis in cardiovascular injury. Acute and chronic infection induced by gram-negative sepsis and viral pathogens (e.g., SARS-CoV-2) trigger canonical and noncanonical inflammasome pathways in myeloid and endothelial cells, leading to IL-1β/IL-18 release and gasdermin-D–mediated pyroptosis. These processes promote immunothrombosis, coagulopathy, and myocardial injury. Chronic immune activation in autoimmune diseases (SLE, RA, psoriasis) sustains vascular inflammation and atherogenesis via NLRP3/AIM2 signaling. In ischemia–reperfusion, cardiomyocyte-derived DAMPs further amplify inflammasome activity, enlarging infarcts. Collectively, this inflammasome–pyroptosis–coagulation axis represents a central pathological hub and therapeutic target for preventing immune-driven cardiovascular events. SLE, systemic lupus erythematosus; RA, rheumatoid arthritis; HMGB1, high mobility group box 1.

At the organ and cellular-network levels, pathogen- and damage-derived cues converge on inflammasomes through shared damage-associated molecular pattern (DAMP) pathways. Early after reperfusion in patients following Myocardial Infarction and Heart Transplantation, dying and distressed cardiomyocytes release ATP, mitochondrial DNA, and HMGB1, which activate NLRP3/AIM2 in infiltrating myeloid cells, amplify inflammation, and enlarge infarcts (138, 139). Animal studies support a time-sensitive intervention window: inhibiting NLRP3 or its effectors within hours of ischemia–reperfusion can reduce infarct size and improve function, whereas delayed suppression may hinder reparative remodeling and scar maturation (138, 140, 141). Collectively, whether initiated by pathogens or sterile injury, immune dysregulation culminates in cardiovascular pathology via the inflammasome–IL-1β/IL-18–pyroptosis axis. Accordingly, stratified, time-aware, and individualized combinations that target upstream triggers (infection control and primary immune disease), midstream platforms (NLRP3/AIM2, GSDMD), and downstream effectors (IL-1 signaling) constitute a rational framework to curb immune-driven cardiovascular injury (**Figure 2**).

## Cross-disease intervention strategies targeting inflammasomes

Since inflammasomes—and particularly IL−1 signaling—play pivotal roles in metabolic, immune, and cardiovascular disease processes, they offer highly attractive therapeutic targets with cross−disease potential. Several intervention strategies targeting this axis have shown promise in comorbid conditions, moving from proof−of−principle trials to early clinical translation (142, 143).

The landmark CANTOS trial tested canakinumab, a monoclonal antibody targeting IL−1β, in post−myocardial infarction patients with elevated high sensitivity C-reactive protein (hsCRP) (144). It demonstrated significant reductions in recurrent cardiovascular events, confirming the link between IL−1β–mediated inflammation and atherothrombosis. However, despite robust lowering of inflammatory markers (IL−6 and hsCRP), canakinumab did not reduce the incidence of new-onset type 2 diabetes, though transient improvement in HbA1c was observed during the first 6–9 months (145). Recombinant IL−1 receptor antagonist therapy (such as anakinra) has improved β−cell secretory function, lowered HbA1c, and reduced systemic inflammation in patients with type 2 diabetes or obesity (146–148). In acute myocardial infarction, short−term anakinra treatment has been associated with smaller infarct size and modest improvements in left ventricular function (149). IL−1 blockade is also well−established in treating autoinflammatory conditions such as cryopyrin-associated periodic syndromes (CAPS), familial Mediterranean fever (FMF), and rheumatoid arthritis (150, 151). IL−18 neutralization is in early trials for Still’s disease and potentially relevant to cardiometabolic inflammation (152).

Moving upstream, selective NLRP3−targeting small molecules such as MCC950 have demonstrated efficacy in preclinical models by binding the NACHT domain to block ATPase activity (153). MCC950 reduces atherosclerosis, stabilizes plaques, ameliorates diabetic nephropathy and cardiomyopathy, and improves insulin sensitivity in animal studies (154, 155). Dapansutrile (OLT1177), a β−sulfonyl nitrile compound with oral bioavailability, inhibits NLRP3 allosterically and has shown safety and anti−inflammatory effects in gout trials (156), with ongoing investigation in heart failure (157). Other candidates—including oridonin, CY−09, tranilast, BOT−4−one, and novel quinazolinone derivatives—are in development (158). Caspase−1 inhibitors (e.g., VX−765) and GSDMD pore blockers such as disulfiram offer alternate means to suppress downstream inflammasome activation, though some face limitations due to toxicity or limited efficacy (159). Efforts to target NEK7–NLRP3 interactions or ASC oligomerization further expand the molecular repertoire of inflammasome−directed strategies (33).

Complementary therapeutic approaches include repurposed drugs and lifestyle interventions that modulate inflammasome activity. Low−dose colchicine disrupts ASC speck formation and NLRP3 assembly; clinical trials (LoDoCo2, COLCOT) showed reductions in ischemic events and support its use as adjunctive therapy post−MI (160, 161). Statins indirectly inhibit NLRP3 by reducing the cellular cholesterol load that otherwise triggers inflammasome activation in metabolic diseases (162). Beyond this, Sodium-Glucose Co-Transporter 2 (SGLT2) inhibitors, GLP−1 receptor agonists, and metformin exert anti−inflammatory effects in part by inhibiting NLRP3 activation, promoting autophagy, or activating AMP-activated Protein Kinase (AMPK) pathways (163, 164). SGLT2 inhibitors lower cardiovascular mortality in both diabetic and non−diabetic patients and may suppress NLRP3 via shifts in ketone metabolism (165). The ketone body β−hydroxybutyrate, elevated in fasting or ketogenic diets, directly inhibits NLRP3 activation (166). Meanwhile, exercise training lowers NLRP3 expression in adipose and cardiac tissue (167). Weight loss, dietary fiber, and probiotics reduce endotoxemia and may attenuate inflammasome priming through gut–immune modulation (168).

**Figure 3:**
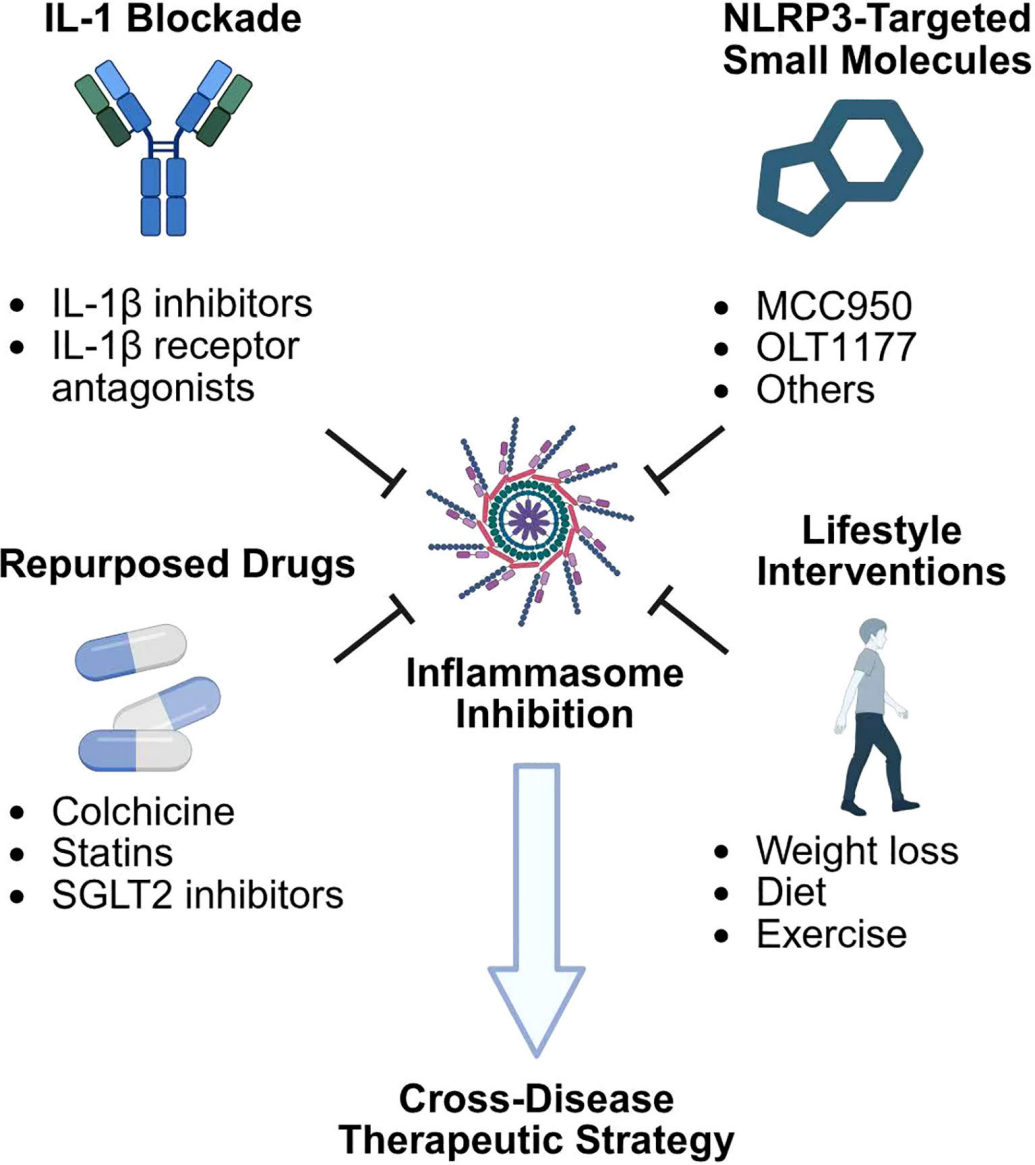
Cross-disease therapeutic strategies targeting the inflammasome–IL-1 axis. IL-1 blockade (e.g., canakinumab, anakinra) reduces inflammation and improves outcomes in atherosclerosis, type 2 diabetes, and autoinflammatory syndromes. Upstream inhibition of NLRP3 (e.g., MCC950, OLT1177) and downstream suppression of caspase-1 or GSDMD limit pyroptosis and cytokine release. Adjunctive approaches—colchicine, statins, SGLT2 inhibitors, GLP-1 receptor agonists, exercise, ketogenic diets—attenuate inflammasome priming and activation through metabolic and autophagy pathways. Together, these strategies illustrate a multi-tiered therapeutic framework with cross-disease potential. IL-1, interleukin-1; SGLT2, sodium-glucose cotransporter 2.

Taken together, these pharmacologic, repurposing, and lifestyle strategies illustrate the translational potential of targeting the inflammasome–IL−1 axis across metabolic, cardiovascular, and immune−mediated diseases. A rational, combinatorial approach—tailored by disease context and timing, with upstream trigger control, direct inflammasome modulation, and downstream cytokine inhibition—appears poised to reshape therapeutic paradigms for comorbid cardiometabolic and inflammatory disease states (**Figure 3**).

## Context-dependent heterogeneity of inflammasomes as the molecular hub

While accumulating evidence establishes the inflammasome as a central molecular hub, a precise theoretical framework requires acknowledging the applicability boundaries of this concept. The role of the inflammasome is not monolithic; rather, it is a highly dynamic and context-dependent modulator of disease processes. Its function as a driver, amplifier, or even a necessary component of a physiological response varies significantly across different pathological conditions and their temporal stages. Understanding this heterogeneity is critical for the rational design of targeted therapies.

The net impact of inflammasome activation is profoundly influenced by the specific disease context. In chronic cardiometabolic diseases, the evidence strongly supports a detrimental role. In atherosclerosis, for instance, the chronic, low-grade activation of the NLRP3 inflammasome within macrophages and endothelial cells is unequivocally pro-atherogenic, promoting lipid accumulation, plaque inflammation, and instability (169). Similarly, in obesity and type 2 diabetes, inflammasome activation in metabolic and immune cells is a key driver of insulin resistance and the systemic low-grade inflammation that underpins cardiovascular complications (62).

In contrast, in other conditions, the role of the inflammasome is more nuanced, and its complete inhibition could be detrimental. The most evident example is in host defense against infection, where inflammasome-driven pyroptosis and cytokine release are essential for clearing pathogens (170, 171). Systemic inflammasome blockade, as seen in clinical trials, can carry a small but significant risk of increased fatal infections, highlighting its indispensable role in immunity (144). Furthermore, in the context of tissue repair, a controlled inflammatory response is a prerequisite for effective healing. Completely suppressing the inflammasome, particularly in the later stages of repair, may impair the necessary clearance of cellular debris and the signaling cascades that orchestrate tissue regeneration and wound healing (172, 173).

Moreover, the functional output of inflammasome activation is profoundly cell-type specific. In macrophages, activation culminates in robust IL-1β/IL-18 secretion, orchestrating a broad inflammatory cascade (174). Conversely, in vascular endothelial cells, the primary outcome of NLRP3 activation is often lytic cell death via pyroptosis, which directly compromises vascular barrier integrity, promotes sustained vascular inflammation and thrombosis (175, 176). Cardiomyocytes, which possess a higher threshold for activation, may undergo pyroptosis that directly contributes to contractile dysfunction and link to the pathogenesis of Atrial Fibrillation (177, 178). Therefore, future therapeutic strategies must evolve beyond pan-inhibition toward spatiotemporally precise modulation, potentially through cell-specific delivery systems or interventions timed to specific phases of disease.

Beyond the specific disease and cell type, the inflammasome’s function is highly dependent on the temporal phase of the pathology, a dynamic best exemplified by the distinct stages of MI. In the acute phase following ischemia-reperfusion, massive DAMP release triggers robust inflammasome activation that is predominantly detrimental. This drives cardiomyocyte pyroptosis and expands the infarct size, meaning early therapeutic inhibition at this stage is strongly cardioprotective (15, 140). During the subsequent inflammatory phase, the inflammasome-driven release of cytokines like IL-1β is essential for orchestrating a necessary clean-up operation. It is critical for recruiting neutrophils and macrophages to clear necrotic debris and prepare the infarct zone for repair (172). In the final phase, persistent and unresolved inflammasome activation becomes maladaptive. It promotes chronic inflammation, adverse ventricular remodeling, and the transition to heart failure. A nuanced approach is required as complete long-term blockade could potentially interfere with signals necessary for optimal scar formation (179).

Therefore, the concept of the inflammasome as the molecular hub must be refined with an understanding of its context-dependent heterogeneity. Its outputs are neither uniformly beneficial nor detrimental but are instead tailored by the specific disease, the cell type, and the timing within the disease process.

## Discussion

Translating inflammasome biology into broad clinical interventions remains promising yet challenging. First, inflammasome signaling is highly cell-specific: cardiomyocytes express low baseline NLRP3 and require strong priming, while monocytes are primed to secrete IL-1β rapidly with minimal stimulus (180). It should be noted, however, that IL-1β secretion can also occur via inflammasome-independent pathways in certain settings. For example, recent work in human macrophages showed TLR4 ligation can induce IL-1β release even in the absence of canonical NLRP3 inflammasome activation (181). Thus, systemic NLRP3 inhibition risks impairing host defense while sparing other cells. Furthermore, non−canonical inflammasome mechanisms (caspase−4/5/11) and alternative proteases (e.g., neutrophil elastase) can compensate if NLRP3 is blocked, underscoring innate immunity’s redundancy and plasticity (182, 183). Indeed, sustained inflammasome suppression raises concerns about increased infection risk—severe IL−1β blockade can worsen outcomes in septic patients, even though it has demonstrated safety in stable populations treated with agents like anakinra or canakinumab (144, 184).

Patient heterogeneity presents another obstacle. Genetic polymorphisms in NLRP3, NLRP1, or IL−1β genes influence individual inflammatory responses (185, 186), and not all patients—especially in diabetes—display inflammasome-driven pathology. Current biomarkers, such as CRP or IL−18, provide only imperfect stratification. Advances in single−cell and proteomic profiling may enable the identification of “inflammasome-high” individuals most likely to benefit from targeted therapy. In cardiovascular disease, timing also matters. Animal studies suggest early inflammasome blockade is protective post–myocardial infarction, whereas delayed inhibition may impede tissue healing. Understanding temporal dynamics in humans remains essential.

Mechanistically, critical issues concerning the initiation, modulation, and outputs of inflammasomes are yet to be fully clarified. The contributions of non−NLRP3 inflammasomes (e.g. AIM2 or NLRC4) to cardiometabolic disease are incompletely defined (187). While AIM2 senses nuclear or cytosolic DNA and has been implicated in mouse models of hypertension and aneurysm (188), its relevance in human cardiovascular disease is still unclear. Moreover, both metabolic processes and specific metabolites raise the possibility that metabolic interventions could be leveraged to suppress inflammasome activation. Cellular quality-control processes such as autophagy and its selective form, mitophagy, act as critical negative regulators by removing damaged organelles and inflammasome components, thereby preventing aberrant activation (189, 190). In parallel, distinct immunometabolites such as succinate can accumulate under inflammatory conditions and promote IL-1β production by stabilizing HIF-1α, whereas itaconate, produced by immune cells, has direct anti-inflammatory effects by alkylating and inhibiting NLRP3 itself (191, 192). The crosstalk between adaptive immunity and inflammasomes is also underexplored. Mapping these networks is crucial to developing holistic therapies.

On the pharmacologic front, developing safer and more selective inhibitors remains urgent. MCC950, although highly potent, was discontinued in early phase II trials for treatment of rheumatoid arthritis due to unexpected liver toxicity (182). Newer allosteric inhibitors such as OLT1177 (dapansutrile) have passed phase I trials with favorable safety profiles and are currently in phase II testing for treatment of gout, heart failure, and other inflammatory conditions (156). Complementarily, drug repurposing of drug such as colchicine, anakinra, statins, SGLT2 inhibitors—offers potential pragmatic paths forward. Combination therapies, such as IL−1β blockade alongside statins or SGLT2 inhibitors, may produce additive benefits by targeting both lipid metabolism and inflammasome-driven inflammation.
